# Isolation of Cells from Glioblastoma Multiforme Grade 4 Tumors for Infection with Zika Virus prME and ME Pseudotyped HIV-1

**DOI:** 10.3390/ijms24054467

**Published:** 2023-02-24

**Authors:** Celine Pöhlking, Sebastian Beier, Jan Patrick Formanski, Michael Friese, Michael Schreiber, Birco Schwalbe

**Affiliations:** 1 Department of Virology, LG-Schreiber, Bernhard Nocht Institute for Tropical Medicine, Bernhard Nocht Str. 74, 20359 Hamburg, Germany; 2 Department of Pathology and Neuropathology, Asklepios Kliniken Hamburg GmbH, Asklepios Klinik Nord, Standort Heidberg, 22417 Hamburg, Germany; 3 Department of Neurosurgery, Asklepios Kliniken Hamburg GmbH, Asklepios Klinik Nord, Standort Heidberg, 22417 Hamburg, Germany

**Keywords:** glioblastoma multiforme, human cerebrospinal fluid, oncolytic viruses, pseudotyped virus, Zika virus, HIV-1

## Abstract

This study aimed to isolate cells from grade 4 glioblastoma multiforme tumors for infection experiments with Zika virus (ZIKV) prME or ME enveloped HIV-1 pseudotypes. The cells obtained from tumor tissue were successfully cultured in human cerebrospinal fluid (hCSF) or a mixture of hCSF/DMEM in cell culture flasks with polar and hydrophilic surfaces. The isolated tumor cells as well as the U87, U138, and U343 cells tested positive for ZIKV receptors Axl and Integrin αvβ5. Pseudotype entry was detected by the expression of firefly luciferase or green fluorescent protein (gfp). In prME and ME pseudotype infections, luciferase expression in U-cell lines was 2.5 to 3.5 logarithms above the background, but still two logarithms lower than in the VSV-G pseudotype control. Infection of single cells was successfully detected in U-cell lines and isolated tumor cells by gfp detection. Even though prME and ME pseudotypes had low infection rates, pseudotypes with ZIKV envelopes are promising candidates for the treatment of glioblastoma.

## 1. Introduction

Glioblastoma multiforme (GBM) is the most common and aggressive tumor of the central nervous system. The average survival time of patients with GBM is between 12–15 months [1,2]. The poor prognosis after surgical excision results from tumor recurrence, which is mainly caused by a subpopulation of highly tumorigenic cells, called GBM stem cells (GSCs) [3]. Without a cure, treatment is complicated and primarily consists of surgical removal followed by radiation and chemotherapy [4]. The GSC subpopulation, the so-called “tumor-initiating cells” [3], is responsible for tumor recurrence as they are resistant to therapeutic interventions [5,6,7] and therefore lead to the failure of conventional therapy [8,9,10] and the development of treatments [11,12]. Since GBM is extremely heterogeneous, it is impossible to identify cells that have moved away from the original tumor, even with the best staining and imaging techniques available. This makes safe and complete tumor resection impossible [13].

Successful therapy must target the remaining tumor-initiating cells that cannot be removed by surgery. One strategy is to administer drugs directly at the site of the tumor. Lower-grade gliomas, for example, are characterized by the presence of the mutated enzyme isocitrate-dehydrogenase-1 (IDH-1). The absence of IDH-1 activity makes the cells dependent on a secondary pathway to produce nicotinamide adenine dinucleotide (NAD) by the enzyme nicotinamide phosphoribosyltransferase (*NAMPT*). Shankar et al. (2018) have developed a system based on a mixed polymer of lactic acid and glycolic acid loaded with an inhibitor for NAMPT [14]. The ultimate aim is to administer the polymer in situ after surgery to release the drug over days or weeks. Such a strategy would allow the administration of NAMP-inhibiting drugs with an overall increased toxic potential. Normally, these drugs would cause severe side effects if administered intravenously. Shankar et al. reported that they had not observed such negative side effects when applying the NAMPT inhibitor to tumor sites in a murine animal model [14].

Besides NAMPT, enzymes of the metabolic pathway are perfect targets to inhibit tumor growth. These strategies are based on drug toxicity, with or without genotyping tumor cells [14,15]. One aim of genotyping strategies is the identification of mutated genes in GBM tumor cells that play a role in positive selection. Parallel mutations are DNA nucleotide substitutions that occur at the same gene location in tumor cells from different GBM patients. Such parallel mutations occur in low-grade gliomas for IDH1 as previously described, but can also be found in other genes for proteins like EGFR (epidermal growth factor receptor) [16], TP53 (human tumor protein 53) [17], PTEN (Phosphatase and tensin homologue deleted on chromosome 10) [16], and RB1 (retinoblastoma tumor suppressor) [16]. In addition, any mutation, such as silent mutations or mutations outside of genes, can be used to identify parallel mutations. Such mutations may not be relevant to the change in cellular phenotype, but they can be used as targets in a genotype-targeted approach to inhibit or kill tumor cells. Since these mutations distinguish GBM tumor cells from normal brain cells, a CRISPR/Cas9 (Clustered Regularly Interspaced Short Palindromic Repeats/CRISPR associated protein 9) guiding RNA (gRNA), designed for one of these mutations, could inactivate these tumor cells. CRISPR/Cas9 “gene scissors” can bind to short sequence motifs on genomic DNA and cut double-stranded DNA at a site defined by the target sequence present in the gRNA molecule. This system can be used to inactivate genes because the cellular DNA repair mechanism will introduce mismatches, rendering them inactive. For this purpose, a special vector is needed to transfer the CRISPR/Cas9 system into the target cells.

Human immunodeficiency virus type-1 (HIV-1) genome-based lentiviral vectors can be used to transfer the whole CRISPR/Cas9 system into eukaryotic cells by using artificial HIV-1 particles, called HIV-1 pseudotype. Pseudotyping is a technique for producing virus particles with a viral envelope that is not derived from the virus used to produce the virus core. An envelope commonly used for pseudotyping HIV-1 is the glycoprotein G from the vesicular stomatitis virus (VSV). VSVg pseudotyped HIV-1 is often used because almost all cells, including brain cells, are permissive to VSV or VSVg pseudotypes [18]. Interestingly, GBM stem-like cancer cells express higher levels of the Zika virus (ZIKV) receptor molecules Axl [19,20,21] and integrin αvβ5 [22,23]. HIV-1 pseudotypes, loaded with the two ZIKV envelope proteins, prM and E, showed infectivity for two glioma-derived cell lines, U87 and 86Hg39 [24]. In particular, the infection experiments using a lentiviral plasmid containing the firefly luciferase reporter gene have shown that HIV-1 genomes cleared of HIV-1 viral proteins (gag, pol, and env) can in principle be used for pseudotyping and gene transfer into glioma cells. However, only two cell lines were used in these studies. Therefore, it is an important task to show that fresh tumor cells isolated directly from tumors can, in principle, be infected with the ZIKV-HIV-1 pseudotype.

For the pseudotype infection experiments, we collected tumor samples from GBM patients with IDH-1 wild-type and isolated cells from these tumors. There are currently no standardized methods for in vitro cultivation of tumor cells [25]. Therefore, we have developed a cell culture method for pseudotype infection studies using only human cerebrospinal fluid (hCSF) or hCSF as a supplement to the standard cell culture medium. Cerebrospinal fluid provides the brain with nutrients for proper neural functions and growth [26,27], and the use of standard or adapted cell culture media could compromise the functionality of isolated tumor cells [28]. For fast and efficient isolation of tumor cells, we also tested different surfaces to which the cells could adhere. Pseudotypes were generated by transfection of COS-1 cells with plasmids for the ZIKV envelope prME, HIV-1 gag, and pol genes, and for the HIV-1 viral genome. To monitor infection of the prME (Z3) pseudotype, firefly luciferase (Luc) or green fluorescence protein (GFP) was used as a reporter expressed from the viral genome. Additionally, we constructed a new ZIKV ME (Δpr) envelope lacking the pr part of the prME envelope. The corresponding Z3- and Δpr-HIV*luc* and HIV*gfp* pseudotypes successfully infected standard glioma cell lines and the freshly isolated tumor cells.

## 2. Results

### 2.1. Isolation of GBM Cells from Tumor Samples

Tumor cells were isolated from tissue samples and transferred directly into cell cultures after the surgical removal of the tumor. All patients were diagnosed with isocitrate-dehydrogenase-1 (IDH-1) wild type and diagnosed positive for Glioblastoma multiforme.

Inhibition of the MGMT gene by methylation of the promotor leads to reduced O-6-methylguanine-DNA methyltransferase expression and reduced DNA repair activity. This may be relevant since such tumors have an increased sensitivity to therapeutic interventions. The MIB-1 labeling index indicates how many cells from a biopsy were mitotically active before surgery. Glial fibrillary acidic protein (GFAP) is a protein found specifically in brain cells and is not found outside the central nervous system (CNS). This protein is only released outside the CNS after cell death or brain injury and is therefore an additional diagnostic marker for glioblastoma multiforme. Most patients have received therapy according to the Stupp protocol [4] before surgery.

Four tumor samples were collected from each of the twelve patients P01-P12 between June and September 2021. The tumor samples were found to be heterogeneous, as differences in growth and appearance of adherent cells were observed between patients and between the four tissue subsamples taken from each tumor. From each of the tumor samples, we obtained a mixed cell suspension using a 70 µm cell strainer.

The cell suspensions were each divided into three portions and cultured in DMEM, 1:1 hCSF+DMEM, and hCSF. The first appearance of adherent cells was monitored between days one and four, and non-adherent cells were removed when the density of adherent cells reached 40–50%. Replication increased as cells began to connect with each other through newly developed long filopodia. The 20-day growth rates of our tumor cell cultures are shown in Table 1.

**Figure 1:**
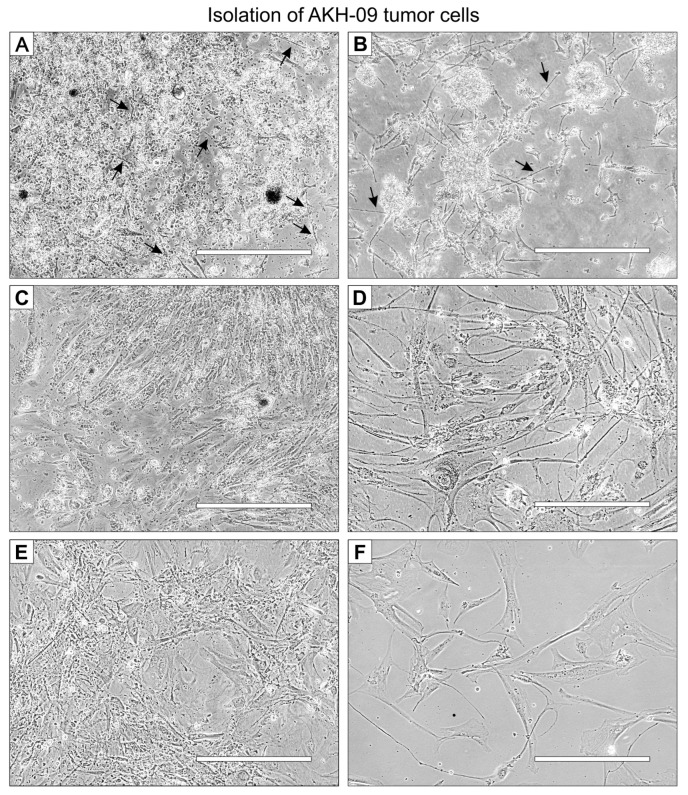
AKH-09 tumor cell isolation at different stages of development. Cells were cultivated in 1:1 hCSF/DMEM with 10% FBS in a T-25 Cell+™ culture flask. (**A**), the whole, mixed cell suspension on day one. (**B**), the whole cell suspension on day two. (**C**), the cell culture cleared of non-adherent cells on day 20. (**D**), the cells from the first subculture were used for infection assays. (**E**), third subculture. (**F**), cells grown from frozen stocks from the third subculture. Black arrows indicate the first appearance of adherent cells. White bars = 400 μm.

A representative example of the isolation of cells from tumor tissue samples is shown for the tissue sample P-09, designated AKH-09. The tumor cells in this example were cultured in a 1:1 mixture of hCSF and DMEM with 10% FBS (FBS final concentration of 5%). On the first day, small, needle-shaped cells appeared (Figure 1A), which developed into a much longer shape within the next few days, surrounded by small non-adherent cells (Figure 1B). Adherent cells developed long filopodia, and lamellipodia began to grow (Figure 1C). From the first subculture, cells were seeded on 96-well plates for infection assays (Figure 1D). The visual phenotype of the isolated cells remains stable in the third subculture (Figure 1E) and cultures started from frozen stocks (Figure 1F).

The cell shape and growth pattern varied significantly between the individual tissue samples. A common feature was the formation of distinct filopodia, which were typically 50–300 µm long (Figure A2). When cells began to make cell-to-cell contact, the filopodia became thicker, while others regressed, and cell replication increased significantly. Tumor cells with cell-to-cell contacts usually form only two or three filopodia, which contact cells in the immediate vicinity and form a meshed, netlike structure, or they form large, triangular clusters of adherent cells, leaving the center of the cluster empty. The isolated cells used for pseudotype infection experiments (AKH-01, -05, -09, -10, -12) were grown on a larger scale, and the corresponding cell pellets were embedded in paraffin wax for standard p53 and S100 tumor marker diagnostics (Figure A3). P53 and S100 detection was <10% in the cultured samples, with two exceptions: AKH-09 and -10, where 80% and 50% of the cells were positive for p53, respectively.

### 2.2. Detection of ZIKV Receptors Axl and Integrin αvβ5 on Cells Used for Pseudotype Infections

**Figure 2:**
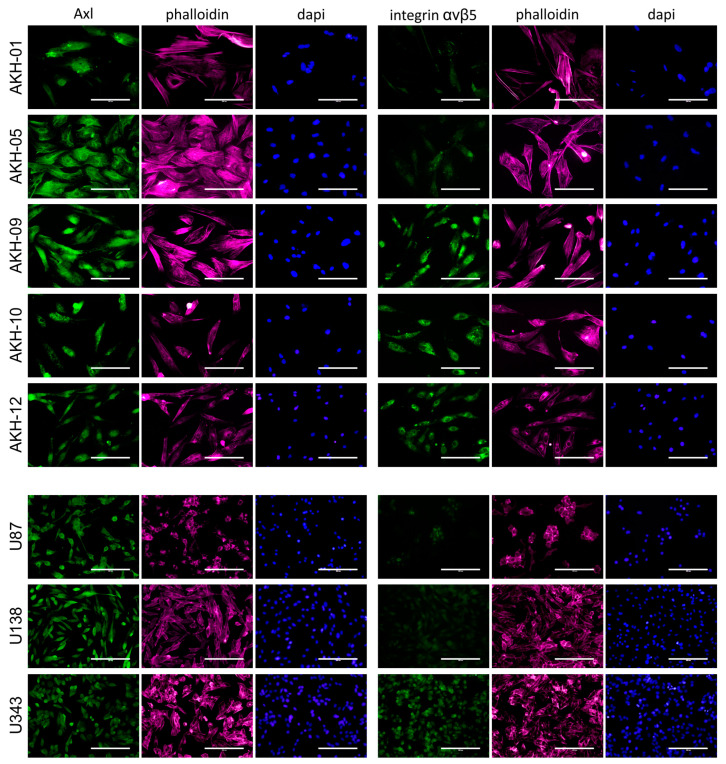
Axl and integrin αvβ5 expression in AKH tumor cells and glioma cell lines. Cells were cultured on glass slides, fixed with formalin, and then post-treated with Triton-X100. Phalloidin (red) and DAPI (blue) were used to visualize the cells and their nuclei. Bound Axl- and integrin αvβ5-specific monoclonal antibodies were detected by using a secondary anti-mouse antibody-Alexa 488 conjugate (green). Scale = 200 µm.

ZIKV receptors were detected using Axl- (mAb clone C4A8) and integrin-αvβ5-(mAb clone P1F6) specific monoclonal antibodies, as shown in Figure 2.

All cells tested were positive for Axl. In addition to these cells, we also tested our standard laboratory cell lines COS-1, VeroB4, and HEK293T for Axl expression. Axl was positive in all these cells, but they were clearly negative for integrin αvβ5. The overall expression of integrin αvβ5 was also very low in AKH-01 and AKH-05, and only a subset of the cells was positive. A very low level of integrin αvβ5 was detected in U87 and U138. AKH-09, -10, -12, and U343 showed similar levels of integrin αvβ5 as Axl.

**Figure 3:**
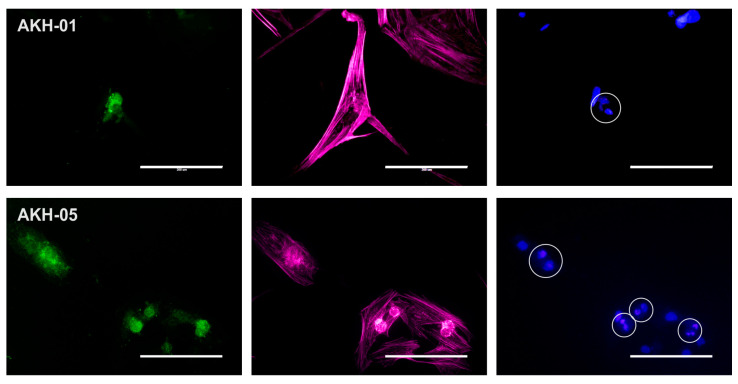
Axl and integrin αvβ5 expression in AKH-01 and AKH-05 tumor cells. Integrin αvβ5 (green) was detected in the center of the cells (AKH-01) and at sites corresponding to the bright round spots in the cell center (AKH-05, light red). At the same time, two nuclei are localized at these sites (blue). Scale = 200 µm.

As mentioned above, we detected low overall integrin expression in both AKH-01 and AKH-05 cell cultures. Figure 3 shows that the low expression of integrin αvβ5 can be seen in the silent cells. Cells in their late dividing state show spots of high integrin αvβ5 expression.

### 2.3. Infection of Glioma Cell Lines and AKH Tumor Cells with ZIKV-HIV Pseudotypes

**Figure 4:**
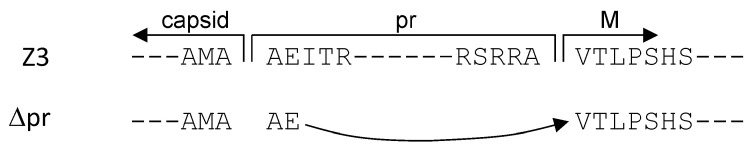
Capsid-pr and capsid-M sequence transition for Z3 and ∆pr, respectively. Z3, ZIKV envelope proteolytic cleavage sites for prME. ∆pr, the AMAAEV sequence joining the capsid to M.

Three HIV-1 pseudotypes with the envelopes Z3 (prME), Δpr (ME), and as a control, VSVg, were used for the infection experiments. The amino acid sequences of the ZIKV capsid-to-pr and capsid-to-M transitions are shown in Figure 4.

**Figure A1:**
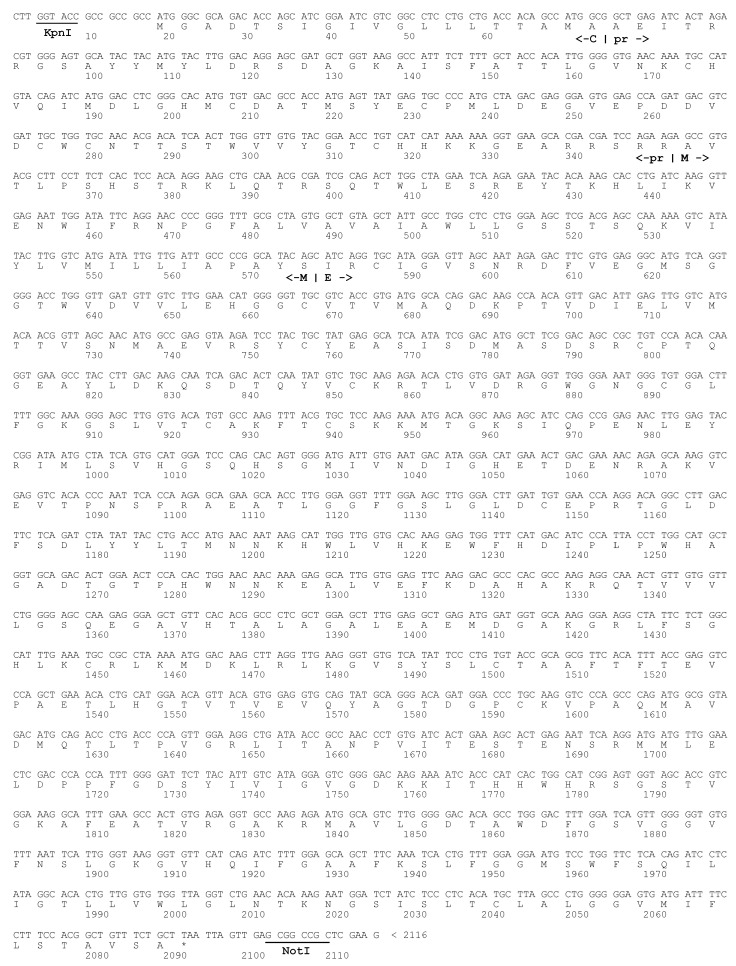
Z3 prME sequence. C, ZIKV capsid c-terminal transmembrane region. prM and E, and ZIKV envelope proteins. KpnI and NotI are restriction sites for cloning into the pME expression vector.

**Figure A2:**
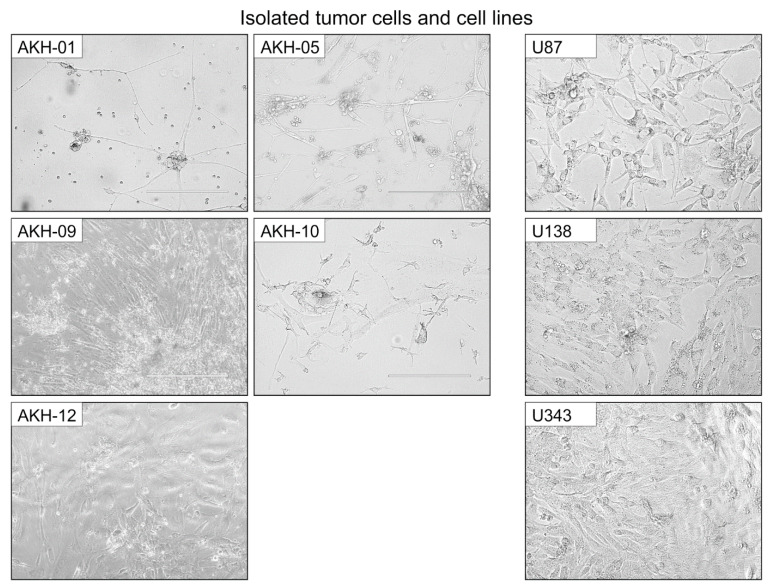
Examples of shapes and growth characteristics from isolated tumor cells and cell lines. AKH, isolated tumor cells, and cell lines (U87, U138, and U343) were used for infection experiments with ZIKV-HIV pseudotypes. AKH-05, AKH-10, and AKH-09 cells were cultured in 1:1 hCSF/DMEM 10%FBS; AKH-01, U87, U138, and U343 cells were cultured in DMEM 10%FBS; and AKH-12 was cultured in 100% hCSF. White bars = 200 μm.

**Figure A3:**
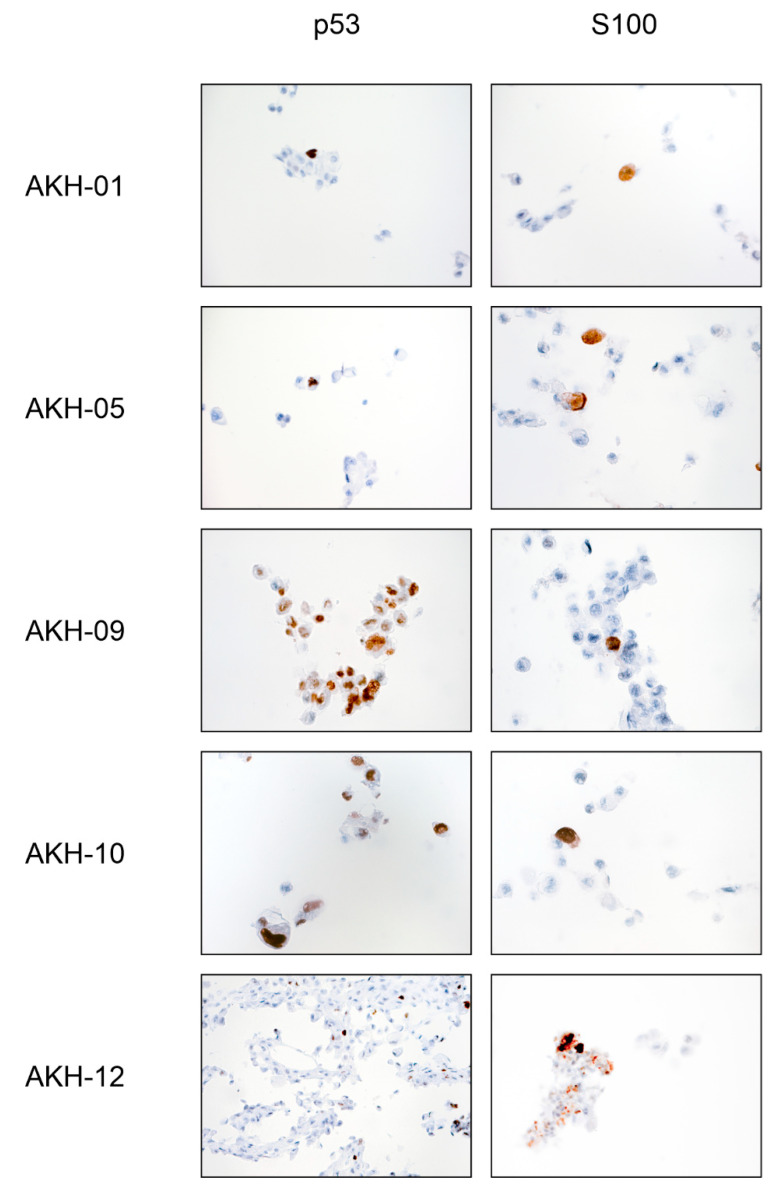
Tumor antigen p53 and S100 staining of isolated AKH tumor cells used for pseudotype infection experiments.

The complete prME sequence is shown in the Appendix A in Figure A1. The amino acid recognition site for proteolytic cleavage AMAAEI of the capsid-to-pr transition was retained in the prME expression vector. For the preparation of the capsid-to-M construct, the AMAAE sequence was coupled to the VTLPSHS start of the M domain, creating an AMAAEV recognition site. Using these two ZIKV envelope constructs, HIV-1 pseudotypes with firefly luciferase as entry reporters were produced using vectors pNL*luc*AM.

**Figure 5:**
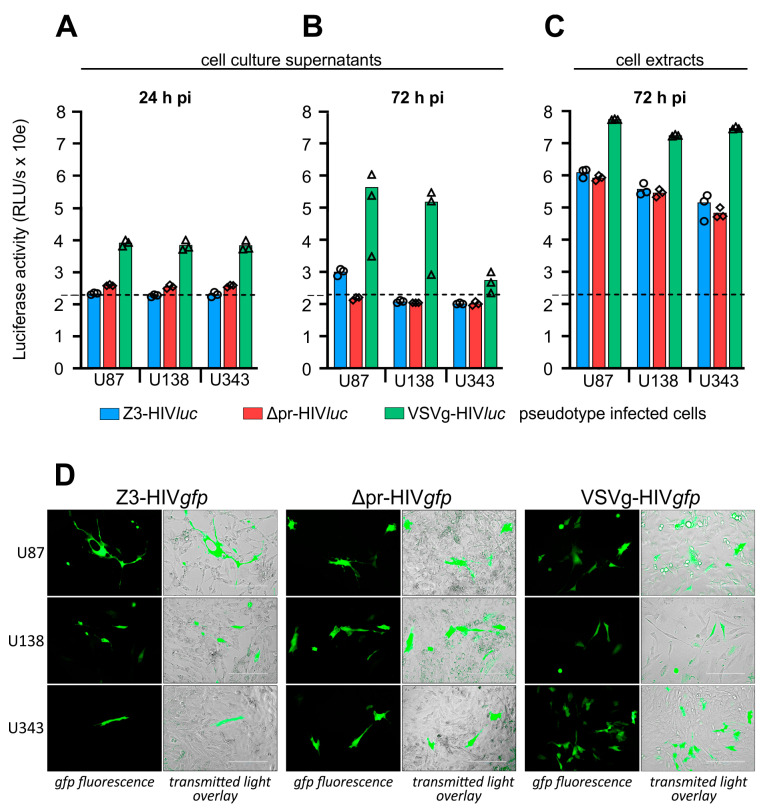
Infection of Glioma cell lines by pseudotyped HIV particles. (**A**), extracellular firefly luciferase activity in cell culture supernatants 24 h post-infection. (**B**), extracellular luciferase, 72 h post-infection, shortly before cell lysis. (**C**), intracellular luciferase tested 72 h post-infection. (**D**), cells were infected with HIV-1 pseudotypes using gfp as a reporter to demonstrate single-cell infection. (**A**–**C**), small symbols show the individual measured RLU/s values. (**D**), Scale = 200 µm.

For infection of glioma cell lines U87, U138, and U343, cells were seeded in 96-well plates and infected using cell culture supernatants containing the pseudotype particles VSVg-HIV*luc*, Z3-HIV*luc*, and Δpr-HIV*luc*. After pseudotype infection, firefly luciferase activity in the culture supernatants was low 24 and 72 h post-infection. Thus, even shortly before the lysis of the cells, no firefly luciferase was measured in the cell culture supernatants. The 24-h values represent the luciferase activity derived from the initial COS-1 transfection supernatants. The 72-h values represent the luciferase activity released during HIV genome integration and subsequent expression (Figure 5A,B). Only the VSVg-HIV*luc* infected cultures had a higher luciferase background in their supernatants, which was most likely due to the weak syncytia-inducing and cell lysis effects observed in these experiments. In all infection experiments, firefly luciferase activity in the cell extracts was 2–3 log10 higher than the so-called background activity detected in the supernatants tested at 24- and 72-h post-infection (Figure 5C).

We also produced HIV-1 pseudotypes with the pNL*gfp*AM vector to show infections of single cells by their green fluorescence (Figure 5D). Compared to VSVg-HIVgfp infection rates, which were estimated at 5–9%, infection rates for Z3- or Δpr-HIVgfp were basically lower, ranging between 0.1 and 0.5%. The best results were obtained with U138 cells, showing an estimated infection rate of about 0.5% for Δpr-HIVgfp.

Both infection experiments using *luc* or *gfp* as entry reporters showed that the ME envelope lacking the pr domain can promote entry into glioma cell lines.

**Figure 6:**
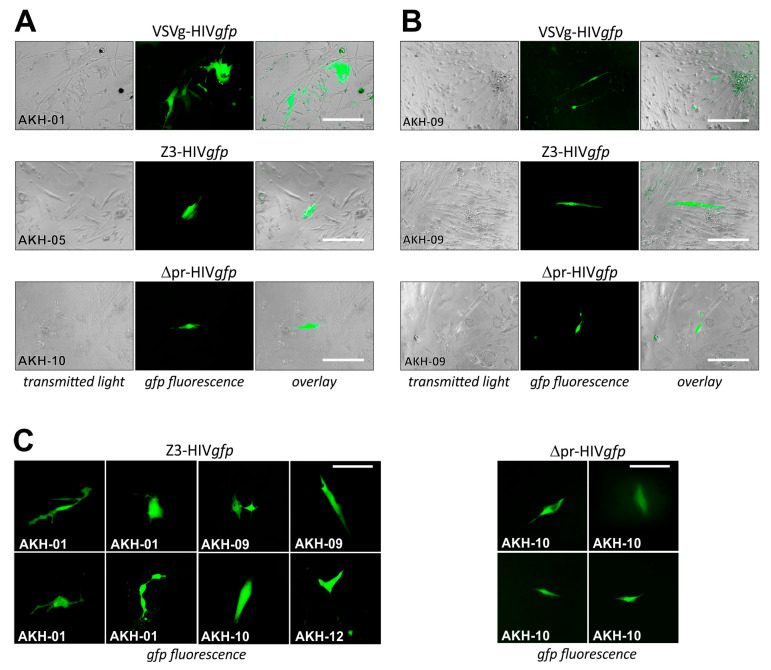
Cells isolated from grade 4 Glioblastoma multiforme tumors infected with ZIKV-HIV*gfp* pseudotypes. (**A**), different AKH-tumor cells infected with VSVg-, Z3-, and ∆pr-HIV*gfp*. (**B**), infections of AKH-09 cells. (**C**), examples of cells infected with pseudotypes Z3-HIV*gfp* and ∆pr-HIV*gfp*. Green fluorescence was monitored at 24 h intervals. Scale for A, B = 200 µm; for C = 100 µm.

For pseudotype infection of AKH tumor cells, these were seeded into Cell+™ 96-well plates and infected using respective cell culture supernatants of COS-1 transfected cell cultures containing VSVg-HIV*gfp*, Z3-HIV*gfp,* and Δpr-HIV*gfp*. Pseudotype entry was monitored by green fluorescence, as shown in Figure 6.

COS-1 cells transfected by pNL*gfp*AM and pCMV-VSVg developed gfp-positive syncytia, indicating that both plasmids within the transfected cells were productive. In Figure 6A, infections are shown for AKH-01, -05, and -10, and in Figure 6B, infections are shown for AKH-09 cells. Compared to VSVg-HIV*gfp* infections, cells in Z3-HIV*gfp* and ∆pr-HIV*gfp* infection experiments showed partial detachment (Figure 6B). As expected from the Z3-HIV*luc* experiments shown in Figure 4A–C, the number of Z3- and ∆pr-HIV*gfp*-related gfp-positive cells were significantly lower compared to VSVg-HIV*gfp* infections in AKH-01 cells. In Figure 6B, data from AKH-09 infections showed that the infection rate for Z3-HIV*gfp* was about one-third that of VSVg-HIV*gfp*. In Figure 6C, more examples of Z3-HIV*gfp* and ∆pr-HIV*gfp* single-cell infections of AKH tumor cells were exemplarily shown.

## 3. Discussion

### 3.1. Isolation of Tumor Cells from Glioblastoma Multiforme Grade 4 Tissue Samples

For the pseudotype infection studies cells were isolated from Glioblastoma multiforme tumor samples and cultivated to establish cell cultures of adherent cells, designated AKH cells. Usually, DMEM and Neurobasal media are suitable for the in vitro cultivation of tumor cells [29]. Considering that DMEM with FBS addition is used in cell culture for rapid cell division but is not developed for post-mitotic cells such as neurons, the lack of proteins and ions required for optimal neuronal growth is one reason [25]. Furthermore, the presence of serum can induce neural stem cell differentiation [27]. Therefore, neurobasal media with various additives have been developed to ensure long-term survival as well as low cellular differentiation during in vitro cultivation [30]. However, several studies have shown that DMEM is often used as a basic medium with or without selected additives for the cultivation of neuronal cells. One possible reason is that DMEM/FBS is much cheaper than commercially available neurobasal media with expensive additives. Due to the great heterogeneity of GBM tumor cells, the choice of media primarily depends on the scientific task [25]. The main methodological objective of the present study was to obtain a high yield of adherent tumor cells that could be used for infection experiments as soon as possible.

A recent study investigated the use of human cerebrospinal fluid (hCSF) as a sole medium or as a medium additive [28]. In the first weeks after the isolation of tumor cells, an increase in cell growth was observed compared to the use of DMEM (10% FBS) medium. Tumor cell proliferation was significantly increased in the presence of hCSF. Thus, the average density of the tumor cell population was reached more quickly. Bardy et al. (2015) also reported that a medium enriched with hCSF components supports basic cellular functions and the overall activity of human neurons [31]. This supports the assumption that hCSF provides the brain with a variety of important nutrients, enabling proper neuronal functions [26,27]. According to a report by Reiber et al. (2001), important proteins needed for cell growth are provided by hCSF [30,32], suggesting that hCSF would be an appropriate supplement to DMEM or Neurobasal media or could be used as a sole medium for the first phase of cell isolation. This is contrary to the often-stated necessity for a full-fledged and thus expensive culture medium, to establish a universal method for the isolation and long-term cultivation of GBM tumor cells [29].

In the present study, cultivation in DMEM with hCSF added showed an overall improvement in cell growth and tumor cell isolation during the first weeks of cultivation. However, when complete cell confluence was achieved, and cells were passaged 2–3 times, the cells adapted to the Cell+™ surface. As a result, after successful isolation, the tumor cells could be grown at the same rates in sole DMEM (10% FBS without hCSF). We, therefore, recommend the use of hCSF as a supplement or sole medium as the preferred starting medium for the isolation of GBM tumor cells for infection experiments with oncolytic viruses or viral pseudotypes.

Another important factor for the isolation of tumor cells is the specific surface characteristics of the cell culture flasks [33]. To determine the best surface for adherent GBM cell growth, Sarstedt Cell™ (red cap), Cell+™ (yellow cap), Eppendorf CCC-FN1-coated surfaces, and surfaces coated with an extracellular matrix (ECM) created by 86Hg39 cells were used. The standard Cell™ polystyrene surface (Sarstedt Red Cap) was optimal for the growth of Glioma laboratory cell lines U87, U343, and U138. For these cell lines, no differences were observed between the different surfaces tested. In contrast, when cells were isolated from tumor tissue, a major difference in cell growth was observed. No adherent cells could be detected on the Standard Cell™ surface for more than 4 weeks. Therefore, the standard Cell™ polystyrene surface was an inappropriate surface for the selection of adherent cells. We then tested the Cell+™ surface (Sarstedt, yellow cap). The Cell+™ culture flasks are coated with polar groups in addition to the standard hydrophilic surface to mimic an in vitro environment that allows the adhesion of so-called fastidious primary cells [34]. Although Sarstedt does not provide information about the exact coating, it states that surface irradiation generates polar amino groups, which provide a closer resemblance to the in vivo microenvironment. For this study, we compared cell growth on different surfaces like Cell+™, ECM-coated, and fibronectin-coated. As mentioned earlier, in this study, cultivation on the Cell+™ surface was the most efficient and economical method for isolating tumor cells.

To create a surface that mimics the microenvironment of Glioma cells, the standard surface of the cell culture flask can be ECM-coated [35]. We used the glioma cell line 86HG39 for the coating. AKH-01 through AKH-05 tumor cells were additionally cultivated on ECM-coated flasks and 24-well plates. The cultivation experiments showed neither recognizable differences nor any benefit compared to cellular growth on Cell+™ flask or 24-well plates. An important argument against this method was that tumor cell cultures can be accidentally contaminated by the 86HG39 cells used for the ECM-coating. Such contamination must be carefully avoided in rare infection events in the isolated tumor cells to be monitored. However, due to the more complicated procedure to produce the ECM-coating, Cell+™ flasks were still used for cultivation.

The growth of AKH-05, -09, and -12 was also tested on Eppendorf CCC-FN1 24-well plates. Tumor cells grew particularly well on these surfaces, but the price difference and small cultivation area (available only in 24-well format) were negative aspects. In general, therefore, no advantage over the Cell+™ surface was observed by using the fibronectin-FN1-coated plates. FN1 plates mimic the cell attachment site of a native extracellular matrix and ensure passage over twenty-five passages without surface-induced cell differentiation [36]. In our experience, FN1 plates were well-suited for the isolation of tumor cells. Unfortunately, FN1 plates are no longer available, and since such coated plates are expensive, we did not search for an equivalent FN1 replacement.

In summary, cell culture plasticware with the Cell+™ surface in combination with DMEM/FBS supplemented with hCSF, or hCSF alone was the method of choice to isolate and culture adherent cells from GBM tumor samples for our pseudotype infection studies.

### 3.2. prME and ME Pseudotyped HIV-1 Particles for Tumor Cell Infections

We previously demonstrated that VeroB4 and two cell lines derived from brain tumors, U87 and 86HG39, can be infected with four different prME-HIV-1 pseudotypes [24]. Based on these studies, we decided to use the prME pME-Z3 expression vector. The Z3 prME envelopes showed high infection rates for U87 and 86HG39 cell lines compared to Z2 and Z4. Since firefly luciferase, which was used as a reporter to detect pseudotype infection in our previous study, is not a suitable reporter to study infections of single cells, we now performed experiments expressing intracellular gfp.

The present study provides further evidence that ZIKV-HIV pseudotypes could be a promising candidate for virotherapy targeting gliomas. In addition to the infection of cell lines, single-cell infections of isolated cells from tumor samples are another important proof of concept. Another important point is that the study clearly focused on cells from GBM tumors, which are highly malignant and resistant to treatment, even after the main tumor has been surgically removed. A therapeutic approach by Shankar et al. (2018) targets the remaining cells, aiming not to harm healthy tissue [14]. This approach will apply only to brain tumors showing the IDH-mutated phenotype and is therefore not suitable for tumors with wild-type, functional IDH-1. This is important because grade 4 GBM tumors all express functional IDH-1, and remaining GBM tumor cells can therefore not be inhibited by the Shankar approach.

However, tumor samples from GBM are generally heterogeneous [33,37]. Due to the diversity of tumor cells, we collected four different tissue samples from each tumor, suggesting that at least one sample includes enough tumor cells and implying that contaminations that cannot be avoided most likely do not occur in all samples. Since GBM tumor cells do not necessarily show rapid cell growth or some samples did not contain enough tumor cells, it was found that some tissue samples did not provide enough adherent cells (AKH-04, 06, 07, 08) and therefore were not included in the infection studies. Owing to the large differences between samples, cultivation and passage, must be adapted in such a way that, once established, cell-to-cell contact should be maintained to avoid any collapse in cellular growth. Of the forty-eight tissue samples collected, fifteen cell cultures, not cell lines, were established with sufficient growth rates to allow the preparation of test plates. All these cultures were infected by the Z3-HIV*gfp* pseudotype. Consistent with the present knowledge, Axl was detected in all the cells [21], while integrin αvβ5 expression varied significantly. Some evidence suggests that Axl may mediate ZIKV entry into astrocytes [38]. However, Axl is not a universal major receptor for ZIKV since Axl is not required for ZIKV infection of neuronal cells [39] or infections in mouse models [40]. In studies using freshly isolated primary human GBM slices, integrin αvβ5 is an important molecular feature mediating infection. Blocking integrin αvβ5 by antibody attenuated ZIKV replication in these slices [41]. Thus, integrin αvβ5 was identified as an internalization factor that increases the ZIKV permissiveness of glioma cells [23,42]. To date, the precise molecular interactions that determine the individual steps of ZIKV entry into glioma cells have not been fully elucidated. Therefore, it is important to study pseudotype infections in fresh tumor cells.

To our knowledge, the studies using Δpr-HIV*luc* or Δpr-HIV*gfp* are the first examples of successful tumor cell infections with a partially truncated ZIKV envelope complex. The Δpr, ME envelope is mimicking the ZIKV envelope as it appears after proteolytic maturation during virus budding. At this point, we argue that the pseudotype infection experiments with prME and ME serve overall as proof-of-principle rather than definitive therapeutic applications. However, the pseudotype model is a very well-suited method to optimize the ZIKV envelope in terms of its receptor affinity and especially its particle packing efficiency. The development of the Δpr pseudotype is another step toward the optimization of the pseudotype envelope. Further work will show whether the prM envelope protein can be completely omitted and whether only the E protein is required for an infectious pseudotype.

In general, enveloped viruses acquire their envelope through a budding process in which ZIKV and HIV-1 are completely different. For viral particle budding, the envelope proteins must accumulate at the appropriate membrane before the final budding step. Zika virus envelope proteins contain transmembrane (TM) localization signals specific for integration into the membrane of the endoplasmic reticulum (ER), and budding takes place in the ER. In contrast, HIV-1 envelopes and the gag and gag/pol precursors are transported to the cellular membrane, where budding is initiated by linking viral membrane-associated proteins to a process called endosomal sorting complexes required for transport (ESCRT). These two different strategies may explain the low efficiency of ZIKV-HIV pseudotype production, which remains a challenge for achieving high pseudotype yields for flavivirus-HIV in general.

An alternative approach was followed by Liu et al., in which stem and ancor regions of E were exchanged against the TM and cytoplasmic domain (CD) of the VSVg envelope protein. This ZIKV-VSV protein chimera had a kidney-specific binding affinity. Together with a lentiviral vector, efficient gene transfer was observed through the corresponding pseudotype. This suggests that functional HIV pseudotypes for glioma cells can likely be produced using a ZIKV E protein in combination with TM and CD sequences from cell membrane-integrated proteins [43].

Although infection rates are relatively low, ZIKV HIV pseudotypes represent a promising method for the treatment of glioblastoma and targeted gene transfer. Since infection rates are low, quantification is difficult. In positive infection experiments, we have identified between 1–5 gfp-positive tumor cells in about 1000 cells. This is in agreement with the observed differences in the measurements with luciferase as a reporter compared to the VSVg-HIV values. In isolated GBM tumor cells, it is even more complicated to quantify precisely, as the cell cultures are very heterogeneous by nature. Therefore, infection rates cannot be directly compared with rates in cell lines. As shown in Figure 6B, the VSV infection rates for the AKH-09 tumor cells are also very low. In comparison, the rates for ZIKV-HIV are one-third of the VSV-HIV rates. This shows that the different tumor cell cultures differ greatly from each other. However, accurate quantification of infection rates with, e.g., FACS requires much more efficient pseudotypes. As a result, greatly improving pseudotype efficiency is an important future goal.

The experiments of Liu et al. [43], who describe a 100-fold higher efficacy of their E-TM-CD construct compared to VSVg, give hope for the development of a more efficient pseudotype. VSV is also formed at the outer cell membrane like HIV. However, VSVg does not contain the PTAP amino acid motif, unlike the HIV p6 protein. This reveals linking the viral budding complex to the TSG101 protein of the ESCRT machinery. Therefore, many more steps need to be taken to create a ZIKV envelope that, like the HIV-1 proteins, finds its way to the cell surface, is efficiently assembled and is finally released by the cellular ESCRT machinery.

Historically, our experience in developing a working pseudotype protocol is in line with reports that we are currently unable to successfully produce infectious ZIKV-HIV-pseudotypes [44]. In line with these findings, we also failed when using the viral pNL4.3R^-^E^-^ vector (nef^−^). ZIKV-HIV, when using identical vector concentrations, as described by Ruiz-Jimenez et al. (2021), we were again unable to detect infectious particles. Successful pseudotype generation established by Kretschmer et al. (2020) relies preferentially on the use of a nef^+^ viral background [45,46], a high vector concentration for prME expression using a modified version of pcDNA3.1 [47], the additional use of a gag/pol packaging vector, and an appropriate transfection protocol [24]. Since the ZIKV-HIV-pseudotypes infect glioma cell lines as well as freshly isolated cells from GBM tumors, it is a future task to enhance pseudotype efficiency by (i) optimizing the codon usage to enhance expression of the envelope [48], (ii) changing ER localization signals present in the envelope sequence [49], modifying signal sequences for outer membrane localization [48,50], and (iii) creating a link to the ESCRT machinery [51]. Since all these steps seem to take their time, the next achievable aim is to generate more efficient prME-, ME- and probably E-HIV-pseudotype particles targeting genes in freshly isolated GBM tumor cells by the CRISPR/Cas9 system [52,53,54,55,56] using the methodology described in this study.

## 4. Materials and Methods

### 4.1. Tumor Specimen

Twelve patients diagnosed with Glioblastoma multiforme (Glioblastoma, IDH-wild type) according to the WHO classification [57] were included in the study. The study design was approved by PV6041 by the Ethical Commission of the Hamburg Medical Chamber (Ethik-Kommission der Ärztekammer, Hamburg, Germany).

Tumor operations were performed at the Asklepios Klinik Nord-Heidberg (Hamburg, Germany). For cell differentiation during surgery, tumor cells were stained with 5-aminolevulinic acid. After surgery, tissue samples were placed in sterile screw cap tubes with a standard cap (2 mL, Type H, Sarstedt, Nümbrecht, Germany) previously filled with 1.5 mL DMEM supplemented with 10% fetal bovine serum (FBS) (PAN-Biotech, Aidenbach, Germany). Tissue samples were collected from four different tumor regions, and each was placed separately in one of the reaction tubes. The tubes were placed in a 50 mL screw cap tube (Sarstedt, Nümbrecht, Germany) to avoid any contamination during transport. The tissue samples were immediately transported to the cell culture laboratory at the Bernhard Nocht Institute for Tropical Medicine (Hamburg, Germany) to start the cell cultivation procedure immediately.

### 4.2. Preparation of Single-Cell Suspension from Tissue Samples

A sterile cell strainer (70 µm mesh size; Fisherbrand, Schwerte, Germany) was placed on top of a 50 mL sterile screw cap tube (Sarstedt, Nümbrecht, Germany). The tissue sample was pressed through the mesh in a circular motion using a sterile plunger flange from a 2 mL syringe (B. Braun, Melsungen, Germany). During the cell separation procedure, the strainer was rinsed multiple times with 2 mL of DMEM/10% FBS. The tube was filled up to 50 mL with DMEM/10% FBS, centrifuged for 10 min (1500 rpm, RT, Megafuge 3.0R, Thermo Scientific Heraeus, Schwerte, Germany), and cells were resuspended in medium.

### 4.3. Cultivation of Tumor Cells

The prepared cell suspension was cultured in 25 cm^2^ filter cap cell culture flasks (T-25, Cell+™, Sarstedt, Nümbrecht, Germany), each containing 10 mL of (i) human cerebrospinal fluid (hCSF), (ii) a 1:1 mixture of hCSF and DMEM/10% FBS, and (iii) DMEM/10% FBS. Cells were grown in a 5% CO_2_ atmosphere at 37 °C. The cell suspension was monitored daily for the appearance of adherent cells. To change the culture medium from the mixed cell culture (adherent and non-adherent cells), non-adherent cells were transferred from the cell culture flask into a sterile 50 mL centrifuge tube (Sarstedt, Nümbrecht, Germany) and centrifuged for 10 min (1500 rpm, RT, Megafuge 3.0R, Thermo Scientific Heraeus, Schwerte, Germany). The cell pellet was suspended in a new medium and placed back in the cell culture flask containing the adherent cells. When the density of adherent cells reached 40–50%, the cell cultures were washed several times with DMEM/10% FBS to remove non-adherent cells. As cell growth increased, the medium was changed every seven days, or the cells were split into two cultures or transferred into larger 75 cm^2^ cell culture flasks (T-75, Cell+, Sarstedt, Nümbrecht, Germany). To split adherent cells, the medium was removed, and the cells were washed with phosphate-buffered saline (PBS) and treated with 1 mL of trypsin 0.05%/ethylenediaminetetraacetic acid (EDTA) 0.02% in PBS (PAN-Biotech, Aidenbach, Germany). After a 2–3 min incubation at room temperature, the cells were resuspended by up-and-down pipetting using 4 mL of cell culture medium and were finally transferred to a new cell culture flask. Images of adherent cells were taken with a fluorescence microscope (EVOS FL Auto, Thermos Fisher Scientific, Schwerte, Germany).

Various cell culture flasks and 24-well plates with different surfaces were used for the growth of tumor cells. One method for the cultivation of neuronal cells is to use surfaces coated with extracellular matrix (ECM) [17]. Therefore, 86Hg39 cells were cultured to high density in DMEM/10% FBS. The cell lawns were treated with 0.5% triton X-100 (2 mL per 25 cm^2^ flask, 0.2 mL per 24-well) for 30 min and washed with PBS. Cell culture flasks and 24-well plates were further incubated with 0.25 M ammonium hydroxide (2 mL per 25 cm^2^ flask, 0.2 mL per 24-well) for ten minutes. After ammonium hydroxide treatment, the surfaces were washed four times with PBS. The flasks or 24-well plates were stored until use at 4 °C.

Another method describes the cultivation of various types of stem cells using fibronectin (FN) [58] or RGD-peptide-coated surfaces [59,60]. A commercially available surface was used (Eppendorf CCC-FN1, Hamburg, Germany) with a surface coated with synthetic RGD-based motifs. The Cell+™ surface provided by Sarstedt (Nümbrecht, Germany) is promoted as a surface for “sophisticated adherent cells”. The Cell+™ surface is loaded with polar amino groups in addition to the hydrophilic polystyrene surface. Cell growth was measured on day 20 by counting adherent cells and expressed as cells/cm^2^ at + = <40, ++ = 40–160, +++ = 160–280, and ++++ = >280 cells/cm^2^.

### 4.4. Immunostaining

Teflon-coated 12-well microscope slides (Thermo Scientific, Schwerte, Germany) were washed with warm, soapy water followed by normal tap water. The slides were then washed with distilled water, isopropanol, and ethanol. They were then autoclaved. For cell culture, the slides were placed in 92-mm Petri dishes, and approximately 1–2 mL of a fresh cell suspension was added on top of the slides. Then, 10 mL of medium was carefully added, and the Petri dishes were stored at 37 °C and 5% CO_2_.

After the appearance of adherent cells, slides were washed three times in PBS buffer to remove DMEM residues. After air drying, slides were placed in a 3.7% formaldehyde solution (30 min, RT) and washed three times with PBS. Fixed cells were then treated with Triton-X100 (0.1% in PBS) for 15 min at RT, and blocking was performed with PBS/5% BSA for one hour in the dark, followed by three washing steps with PBS 0.05% Tween 20 (PBST). Cells were first stained with phalloidin (Phalloidin-iFluor 555 conjugate, AAT Bioquest, Biomol, Hamburg, Germany). 25 µL of a phalloidin working dilution (1 µL/mL PBS/1% BSA) was used per well. Staining was performed for one hour in the dark.

For receptor staining, the primary antibody against Axl (mouse mAb clone C4A8, Invitrogen, Thermofisher, Schwerte, Germany) or integrin αvß5 (mouse mAb clone P1F6, Abcam, Berlin, Germany) was diluted in PBST/1% BSA at 4 µL/mL or 10 µL/mL, respectively. For staining, 25 µL of the antibody working solution was added to each well, and the slides were incubated overnight at 8 °C in the dark. The slides were washed three times with PBST and incubated with 25 µL of the secondary antibody solution (2 µL goat anti-Mouse IgG H&L-Alexa 488, [ab150117, Abcam, Berlin, Germany]/mL PBST/1% BSA) for one hour at RT in a dark chamber. After washing the cells three times with PBST, the last step was to color them with a commercially available DAPI solution (ROTI Mount FluorCare DAPI, Carl Roth, Karlsruhe, Germany). Images of green, red, and blue fluorescent cells were acquired using a fluorescence microscope (EVOS FL auto imaging system, Thermofisher Scientific, Schwerte, Germany).

### 4.5. Production of HIVluc and HIVgfp Pseudotype Particles

Transfection of cells was carried out as described before in 6-well and 24-well formats [24]. COS-1 cells were transfected with plasmids for the HIV-1 virus core pNL*luc*AM or pNL*gfp*AM, provided by Nikolas Friedrich (Institute for Medical Virology, University of Zürich, Switzerland), a packaging plasmid psPAX2 (Addgene, #12260, Teddington, UK), a Zika prME and ME envelope plasmid (pME-Z3, -Δpr, Figure A1), and a VSVg envelope plasmid pCMV-VSV-G (Addgene, #8454) [19]. For transfection in 25 cm^2^ cell culture flasks (T-75, Cell+™, Sarstedt, Nümbrecht, Germany), COS-1 cells were seeded one or two days before transfection, to reach about 70–80% confluence. For transfection, 480 µL of ScreenFect™ Dilution Buffer and 480 µL of ScreenFectA (SFA) (Screenfect GmbH, Eggenstein-Leopoldshafen, Germany) were mixed in a 15 mL sterile screw cap tube (Sarstedt, Nümbrecht, Germany). In a 1.5 mL reaction tube, 480 µL of dilution buffer and the respective plasmid DNA—(i) 150 µg of the pME-Z3 envelope expression vector, (ii) 60 µg of the psPAX2 packaging vector, and (iii) 30 µg of the pNL*gfp*AM vector—were mixed. The DNA mixture was added to the SFA solution and rapidly mixed with at least ten pipette strokes. After 20 min of incubation, OptiPro serum-free medium (Gibco, Schwerte, Germany) was added to a final volume of 6 mL. The culture flask was prepared by discarding the medium and washing the cell layer once with PBS to remove the medium and FBS. The DNA/OptiPro SFM mixture was carefully added to the cell layer, and the flask was incubated at 37 °C and 5% CO_2_. After three hours, the transfection mixture was discarded. The cell layer was washed with PBS, and 12 mL of DMEM containing 10% heat-inactivated FBS (30 min, 56 °C) was added. The cells were incubated at 37 °C with 5% CO_2._ Transfection efficiency was monitored by fluorescence microscopy (EVOS FL Auto, Thermofisher Scientific, Schwerte, Germany). Pseudotypes with firefly luciferase as a reporter were prepared using pNL*luc*AM instead of pNL*gfp*AM according to this protocol and as described previously [24]. Cell culture supernatant was harvested 72 h post-transfection, and centrifuged at 10,000× *g* for 60 s (Eppendorf, Centrifuge 5415C, Hamburg, Germany). Supernatants were directly used for infection experiments or transferred into 1.5 mL high-speed centrifuge tubes (1.5 mL, microcentrifuge tube, Beckman Coulter, Krefeld, Germany). Centrifugation was carried out at 125,000 g for 4 h at 4 °C (TLA-55 rotor, Optima TL, Beckman Coulter, Krefeld, Germany). Pseudotype particles were directly used or stored for up to 7 days at 8 °C.

### 4.6. Infection of Tumor Cells by Pseudotype Particles

The fetal bovine serum used for infection studies was heat-inactivated at 56 °C, under sterile conditions, for 30 min, shaking the tube every 10 min [33]. After heat-inactivation, the FBS was added to the DMEM medium at a final concentration of 10% (PAN-Biotech, Aidenbach, Germany). Two days before infection, tumor cells were seeded in 96-well plates (Cell+™, Sarstedt, Nümbrecht, Germany) in a total volume of 200 µL of the respective medium (hCSF; 1:1 HCSF/DMEM 10% FBS; DMEM 10% FBS) to reach about 70% confluence on the day of infection. Empty microplate wells were filled with 200 µL of PBS to prevent medium evaporation during cultivation. On the day of infection, the medium was discarded and 100 µL of pseudotype solution was added per 96-well plate. For infection, cells were incubated for 3 h at 37 °C with 5% CO_2,_ and then 100 µL of cell culture medium was added. Cells were incubated for 7 days while infection events were observed at daily intervals by an automated multichannel fluorescence life cell imaging system (EVOS FL Auto Imaging System, Life Technologies, Fisher Scientific, Schwerte, Germany).

## 5. Conclusions

GBM is a highly malignant brain tumor. Treatment usually consists of surgery, but despite maximum treatment with chemotherapy and radiation, the tumor recurs. A variety of potential gene targets have been identified to inhibit GBM tumor cells on a DNA level. The challenge remains to develop the appropriate vectors for successful delivery of genetic tools to hit these targets. Our study describes the development of a vector for gene transfer into GBM cells.

Pseudotyping is a useful technology to study viral entry into cells by an envelope-specific process. The pseudotype transfer of a reporter gene such as gfp into the target cells allows a simple but also precise study of pseudotype entry and gene delivery. We constructed HIV particles that contain the prME or ME envelope of ZIKV.

To study infection by these pseudotypes, a culture method was established using hCSF as a supplement or sole medium to isolate cells from GBM tumors. The isolated tumor cells were successfully infected by pseudotypes, as shown by the expression of the entry reporters gfp and firefly luciferase. Although the infection rates were low, these results provide further insight into the construction and use of ZIKV-HIV pseudotypes as a model for the development of an efficient oncolytic pseudotype or virus with a distinct GBM tropism.

## Figures and Tables

**Table 1:** Clinical parameters of IDH-1 wild-type GBM grade 4 patients and parameters of isolated tumor cells.

Patient	MGMT- Promotor ^1^	MIB-1 ^2^	GFAP ^3^	Pretherapy	Sample Date	Isolated Tumor Cells Growth Rate ^4^ p53 S100		
P-01	not methylated	>40%	positive	none	6/21	**++** ^6^	**<10%**	**<10%**
P-02	hypermethylation	20%	positive	none	6/21	+	nd	nd
P-03	not methylated	12%	positive	Stupp ^5^	6/21	++	nd	nd
P-04	not methylated	>30%	positive	mitoxantrone	7/21	+	nd	nd
P-05	not methylated	30%	positive	none	7/21	**+++** ^6^	**<10%**	**<10%**
P-06	hypermethylation	30%	positive	Stupp	7/21	-	nd	nd
P-07	not methylated	30%	positive	Stupp	7/21	+	nd	nd
P-08	hypermethylation	10%	positive	Stupp	7/21	+	nd	nd
P-09	hypermethylation	40%	positive	Stupp	8/21	**++++** ^6^	**80%**	**<10%**
P-10	hypermethylation	40%	positive	Stupp	8/21	**+++** ^6^	**50%**	**<10%**
P-11	not methylated	>30%	positive	none	9/21	+++	nd	nd
P-12	hypermethylation	20%	positive	none	9/21	**++++** ^6^	**<10%**	**<10%**

^1^ O-6-methylguanine-DNA methyltransferase; ^2^ MIB-1(Ki67) labeling index; ^3^ glial fibrillary acidic protein; ^4^ cell density per cm^2^ on day 20, + = <40, ++ = 40–160, +++ = 160–280, ++++ = >280, - = no growth of adherent cells; ^5^ *Stupp protocol* has become the standard of care for the treatment of glioblastoma [4,9]; ^6^ Isolated cells from these patients were used for pseudotype infection experiments.

## Data Availability

Raw data are given by the photographs. Digital files obtained by microscopy were adjusted only for contrast, intensity, and brightness.
